# Dataset on critical parameters of dispersion stability of Cu/Al_2_O_3_ nanofluid and hybrid nanofluid for various ultra-sonication times

**DOI:** 10.1016/j.dib.2019.01.007

**Published:** 2019-01-09

**Authors:** F.R. Siddiqui, C.Y. Tso, K.C. Chan, S.C. Fu, Christopher Y.H. Chao

**Affiliations:** a Department of Mechanical and Aerospace Engineering, The Hong Kong University of Science and Technology, Hong Kong; b School of Energy and Environment, City University of Hong Kong, Hong Kong; c Building Energy Research Center, Guangzhou HKUST Fok Ying Tung Research Institute, Guangzhou, China; d Department of Mechanical Engineering, The University of Hong Kong, Hong Kong

## Abstract

The data presented in this data article comprises the critical parameters of dispersion stability such as the particle effective diameter, zeta potential, sedimentation velocity and stability factor for Cu/Al_2_O_3_ single particle nanofluid and hybrid nanofluid samples at various ultra-sonication times, that is, 0.5 h, 1.0 h, 2.0 h and 3.0 h. The data for effective diameter and zeta potential was generated using the particle size analyser and zeta potential analyser respectively. The measured data for effective diameter and zeta potential was processed to generate the data for sedimentation velocity and stability factor. The ultra-sonication time with maximum value of stability factor was used for sample preparation of Cu/Al_2_O_3_ single particle nanofluid and hybrid nanofluid in the related research article “On trade-off for dispersion stability and thermal transport of Cu-Al_2_O_3_ hybrid nanofluid for various mixing ratios” (Siddiqui et al., 2019) [1].

## Specifications table

**Table:** 

Subject area	*Thermo-fluid Engineering*
More specific subject area	*Hybrid nanofluid*
Type of data	*Table*
How data was acquired	*Ultra-sonication Bath (Model 2510, Branson, USA), Particle Size Analyser (90 Plus/BI-MAS, Brookhaven Instruments Corp., USA), Zeta Potential Analyser (ZetaPALS, Brookhaven Instruments Corp., USA)*
Data format	*Raw data for effective diameter and zeta potential, analyzed data for sedimentation velocity and stability factor.*
Experimental factors	1. *A thick paste was prepared by adding 3 drops (50 µl each) of deionized (DI) water in a 4 ml glass vial containing nanoparticles.* 2. *The thick paste was diluted with DI water up to 4 ml volume, transferred to a 500 ml glass bottle and further diluted with DI water up to 10 ml volume to obtain a 0.01% volume fraction for ultra-sonication.* 3. *Samples were sonicated in ultra-sonication bath at fixed frequency and power of 40 kHz and 130 W respectively.*
Experimental features	1. *Sample preparation of Cu/Al*_*2*_*O*_*3*_ *single particle nanofluid and hybrid nanofluid using ultra-sonication bath.* 2. *Sample characterization based on particle size and zeta potential analysis.*
Data source location	*The Hong Kong University of Science and Technology, Hong Kong.*
Data accessibility	*Data is with the article*
Related research article	*F.R. Siddiqui, C.Y. Tso, K.C. Chan, S.C. Fu, C.Y.H. Chao, On trade-off for dispersion stability and thermal transport of Cu-Al*_*2*_*O*_*3*_*hybrid nanofluid for various mixing ratios, Int. J. Heat Mass Transf. 132 (2019) 1200–1216,*[1]

## Value of the data

• The data comprises the critical parameters of dispersion stability such as the effective diameter, zeta potential and sedimentation velocity for different ultra-sonication times.
• The data can be used to develop an ultra-sonication time optimization model to achieve a uniform and homogenous particle dispersion.
• The data can be used as a reference material to further investigate the ultra-sonication time based dispersion stability for other nanofluid and hybrid nanofluid samples.

## 1 Data

The data on particle effective diameter (∅), zeta potential (ζ), sedimentation velocity ${(V}_{s})$ and stability factor (***SF***) for Cu/Al_2_O_3_ single particle nanofluid and hybrid nanofluid samples at various ultra-sonication times (***t***) is presented in Table 1. The details on sedimentation velocity and various mixing ratios of hybrid nanofluid (MR-1, MR-2 and MR-3) samples are reported in the related research article [1]. The data on scaling factor ($S_{\zeta }$) as shown in Table 1 was processed from the measured data on zeta potential ($S_{\zeta }=0.01665\zeta$). The data in Table 1 shows that the ultra-sonication time with maximum stability factor is 2.0 h for both Al_2_O_3_ nanofluid and MR-1 hybrid nanofluid and 0.5 h for both MR-2 and MR-3 hybrid nanofluids. Stability factor for Cu nanofluid is not presented in Table 1 due to the lack of data on effective diameter as it was above the measuring range of the particle size analyser.

**Table 1:** Critical parameters and stability factor for different samples and ultra-sonication times.

**Sample**	***t* (h)**	∅**(nm)**	ζ**(mV)**	$S_{\zeta }$	$V_{s}.10^{6}$**(m s^-1^)**	$\mathrm{SF}=S_{\zeta }{/(V}_{s}.10^{6})$**(s m^-1^)**
**Al_2_O_3_**	0.5	243.5	52.18	0.871	0.096	9.04
	1.0	214.5	51.77	0.865	0.074	11.55
	2.0	225.3	58.39	0.975	0.082	11.81
	3.0	226.1	46.46	0.776	0.083	9.33
**MR-1**	0.5	250.3	39.55	0.660	0.152	4.32
	1.0	255.3	41.72	0.697	0.158	4.38
	2.0	257.7	43.84	0.732	0.161	4.52
	3.0	276.6	44.67	0.746	0.186	4.00
**MR-2**	0.5	243.9	40.45	0.676	0.177	3.81
	1.0	263.7	35.81	0.598	0.207	2.89
	2.0	250.8	37.61	0.628	0.187	3.35
	3.0	265.8	46.52	0.777	0.210	3.69
**MR-3**	0.5	241.6	45.63	0.762	0.205	3.71
	1.0	247.4	38.43	0.642	0.215	2.98
	2.0	268.9	44.91	0.750	0.254	2.95
	3.0	255.2	45.35	0.757	0.229	3.30
**Cu**	0.5	> 3000	5.30	0.088	–	–
	1.0	> 3000	5.00	0.083	–	–
	2.0	> 3000	6.26	0.104	–	–
	3.0	> 3000	17.89	0.298	–	–

## 2 Experimental design, materials and methods

The experimental design and detailed methodology of Cu/Al_2_O_3_ single particle nanofluid and hybrid nanofluid synthesis using ultra-sonication bath and characterization using particle size analyser and zeta potential analyser are discussed in the related research article [1].
