# Supplementary material for: Dataset on critical parameters of dispersion stability of Cu/Al2O3 nanofluid and hybrid nanofluid for various ultra-sonication times
Source: Data Brief. 2019 Jan 9;22:863–5. doi: 10.1016/j.dib.2019.01.007 (PMC6348288; doi:10.1016/j.dib.2019.01.007)
Supplement: Supplementary file 1 — Supplementary material [file mmc1.docx]

**Disclosure Statement**

We declare that we do not have any actual or potential conflict of interest including any financial, personal or other relationships with other people or organizations that could inappropriately influence (bias) our work.

**Role of the Funding Source**

The funding for this research is provided by the Hong Kong Research Grant Council via Collaborative Research Fund (CRF) account no. C6022-16G; General Research Fund (GRF) account no. 16202517 and Science and Technology Planning Project of Guangdong Province, China. No. 2017A050506010 and No. 2017A050506014.

.
